# Impact of the ambulatory blood pressure monitoring profile on cognitive and imaging findings of cerebral small-vessel disease in older adults with cognitive complaints

**DOI:** 10.1038/s41371-021-00490-y

**Published:** 2021-02-15

**Authors:** Yong S. Shim, Hae-Eun Shin

**Affiliations:** 1 Department of Neurology, Eunpyeong St. Mary’s Hospital, The Catholic University of Korea, Seoul, Korea; 2 Department of Neurology, Bucheon St. Mary’s Hospital, The Catholic University of Korea, Bucheon, Korea

**Keywords:** Hypertension, Preventive medicine, Risk factors

## Abstract

We investigated ambulatory blood pressure (BP) monitoring (ABPM) profiles and magnetic resonance imaging (MRI) findings of cerebral small-vessel disease (cSVD) in older adults with cognitive complaints who were grouped as follows: subjective cognitive decline, mild cognitive impairment, and dementia of Alzheimer’s type. Group comparisons and correlation analyses among demographic characteristics, cognitive and MRI findings, and ABPM profiles were performed. Furthermore, multivariate logistic regression analyses for dependent variables of (1) dementia or not and (2) MRI criteria of subcortical vascular dementia (SVaD) or not were conducted with independent variables of dichotomized ABPM profiles. A total of 174 subjects (55 males and 119 females) were included: mean age 75.36 ± 7.13 years; Mini-Mental State Examination (MMSE) score 20.51 ± 6.23. No MRI and ABPM findings except medial temporal atrophy were different between three groups. Twenty-four-hour systolic BP (sBP) was correlated with MMSE score (*r* = –0.182; *p* = 0.022) and the severity of white matter hyperintensity (WMH) (*r* = 0.157; *p* = 0.048). A higher daytime sBP was associated with dementia (odds ratio (OR): 3.734; 95% confidence interval (CI): 1.041–13.390; *p* = 0.043) and MRI finding of SVaD (OR: 10.543; 95% CI: 1.161–95.740; *p* = 0.036). Although there were no differences in ABPM profiles between three groups, a higher BP—especially a higher sBP—correlated with cognitive dysfunction and severity of WMH in older adults. Only higher daytime sBP was an independent predictor for dementia and MRI findings of SVaD. Among various ABPM profiles in this study, a higher BP, especially a higher sBP, may be considered the most important for clinical and MRI findings of cSVD.

## Introduction

Cerebral small-vessel disease (cSVD) is caused by a group of pathological processes involving perforating cerebral arterioles, capillaries, and venules of the brain [1]. On magnetic resonance imaging (MRI), cSVD can be observed as lacunae, white matter hyperintensities (WMHs), cerebral microbleeds, and so on [2, 3]. These imaging findings can be seen in cognitively normal older adults as well as patients with dementia, including those with Alzheimer’s disease (AD). Aside from being an important cause of stroke, cSVD is a common vascular risk factor of dementia and a major contributor to dementia mixed with AD [2]. Some studies have suggested that cSVD has an additive effect on cognitive decline in patients with AD. Cognitive impairment associated with cSVD was primarily a result of hippocampal and cortical changes [4], but abnormal white matter volume was independently related to dementia severity in Alzheimer disease [5]. Although the pathogenesis of cSVD is not completely understood, hypertension is a well-known major risk factor [6, 7].

Traditionally, 24-hour ambulatory blood pressure (BP) monitoring (ABPM) has been used to study BP under normal living conditions as it offers a reliable estimate of habitual diurnal BP rhythm, which may be used to independently predict hypertension-related complications [8]. Although average 24-hour, daytime (awake), and nighttime (asleep) BP have been the principal components of the ambulatory BP profile investigated as prognostic determinants, other summary measures exist for describing varying aspects of ambulatory readings, including nocturnal dipping, BP variability, and pulse pressure [9–11]. In normal subjects, the mean nocturnal systolic BP (sBP) is 10–20% lower than the mean daytime sBP, a phenomenon known as dipping [12]. Alteration of this nocturnal dipping is associated with an elevated risk of end-organ injury, particularly to the heart, brain, and kidneys [12–14]. Many studies have found that the degree of nocturnal dipping determines the consequent type of cerebrovascular injury. O’Brien et al. [13] and Staessen et al. [15] reported that the incidence rate of stroke was higher in nondippers than in those with a normal dipping pattern. In addition, high BP variability, defined by a standard deviation (SD) of nighttime sBP of at least 10.8 mmHg, was associated with a significantly greater risk (41%) of cardiovascular events, a greater risk (55%) of cardiovascular death, and an increased risk (59%) of all-cause mortality [16].

However, the few studies that have been published to date on the relationship between ABPM profile, cognition, and cSVD offer conflicting results. Some studies have suggested that the severity levels of cSVD and cognitive dysfunction are affected by loss of nocturnal dipping [17] or by sBP variability [18], whereas others have produced opposing results. This apparent disparity may be due to differences in number of study subjects, heterogeneity of clinical symptoms, and methodologies and assessment techniques applied [19, 20].

We investigated the ABPM profiles and MRI findings of cSVD in older adults with cognitive complaints who were grouped into the following three groups: subjective cognitive decline (SCD), mild cognitive impairment (MCI), and dementia of Alzheimer’s type (DAT) [21]. We examined which ABPM profiles have an influence on cognitive function and the imaging findings of cSVD such as WMHs, lacunae, and cerebral microbleeds.

## Methods

### Subjects

This was a single-center cross-sectional study approved by the Institutional Review Board of Bucheon St. Mary’s Hospital, The Catholic University of Korea in Seoul, Korea. We consecutively enrolled patients who visited the hospital’s Department of Neurology clinic from January 2018 to January 2019. Patients with SCD, MCI, or DAT were recruited.

Petersen’s criteria were used to identify the MCI group, which included patients with an objective memory impairment less than 1.5 SD from the norm in at least one memory test but who were still conducting normal activities of daily living [22]. DAT patients were those who fulfilled the criteria proposed by the National Institute of Neurological and Communicative Disorders and Stroke and the Alzheimer’s Disease and Related Disorders Association [23]. SCD was diagnosed when there was a complaint of memory decline without objective neuropsychological abnormal findings in neuropsychological tests [24, 25]. Comprehensive neuropsychological assessment was conducted with the Seoul Neuropsychological Screening Battery (SNSB), which consists of a digit-span task (forward and backward), the Korean version of the Boston Naming Test, the Rey Complex Figure Test (composed of copying, immediate and 20-min-delayed recall, and recognition), the Seoul Verbal Learning Test (three learning-free recall trials involving 12 words, a 20-min delayed recall trial of these 12 items, and a recognition test), the phonemic and semantic Controlled Oral Word Association Test, and the Stroop test (word and color reading of 112 items over a 2-min period). These tests were administered by trained neuropsychologists. Age-, sex-, and education-specific norms based on 447 normal controls were used to interpret the SNSB results. Scores <16th percentile, which is comparable to –1 SD of the norm, were defined as abnormal. All subjects underwent physical and neurological examinations, blood tests (i.e., complete blood count, blood chemistry, vitamin B12/folate, syphilis serology), thyroid function tests, assessment of global cognitive functioning with the Korean version of the Mini-Mental State Examination (MMSE), MRI and magnetic resonance angiography (MRA) of the brain, and 24-hour ABPM. Subjects were excluded from the study if they presented large territorial infarcts on MRI, were younger than 55 years of age, had brain lesions related to cognition, such as brain tumors, encephalitis, or normal pressure hydrocephalus, or had a major psychiatric disease. Participants with a diagnosis of delirium and those unable to be assessed because of conditions such as blindness and/or deafness were similarly excluded. Finally, individuals with a history of alcoholism or other substance abuse or dependence within the past 10 years were similarly excluded. Inclusion criteria were having undergone both brain MRI and 24-hour ABPM within 1 month, having agreed to participate in the study, and having provided informed consent.

### MRI

**Fig. 1:**
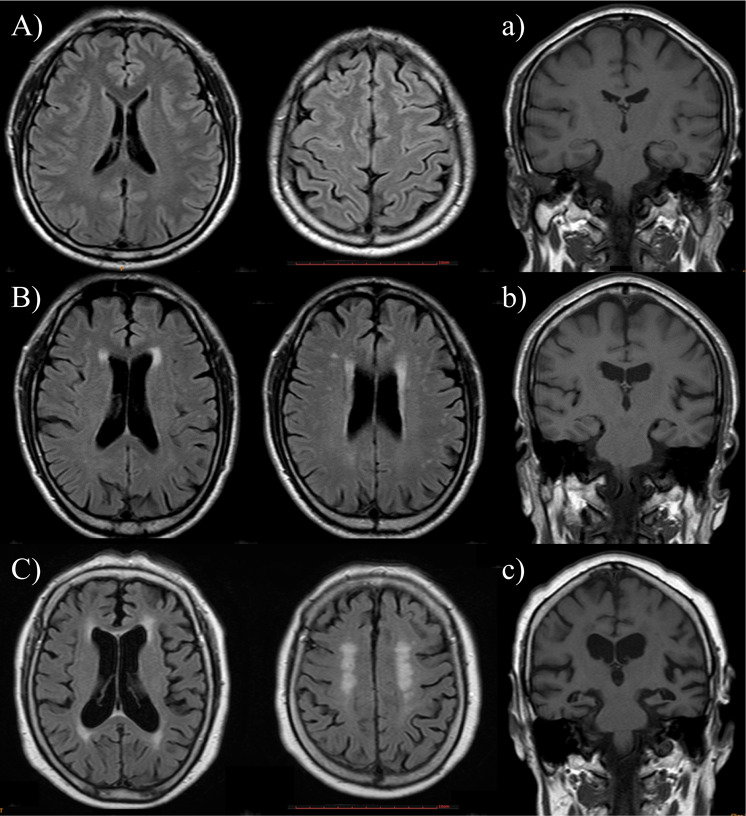
Examples of MRI findings in the three groups, SCD (A, a), MCI (B, b), and DAT (C, c). PVH and DWH were separately evaluated on FLAIR images (**A**–**C**) and MTA was rated on T1-weighted coronal images (**a**–**c**). PVH and DWH were rated as 0 and 0 in SCD, 2 and 1 in MCI, and 3 and 2 in DAT. Right and left sides of medial temporal atrophy were counted as 0 and 0 in SCD, 0 and 1 in MCI, and 4 and 4 in DAT. MRI magnetic resonance imaging, SCD subjective cognitive decline, MCI mild cognitive impairment, DAT dementia of Alzheimer’s type, PVH periventricular white matter hyperintensity, DWH deep white matter hyperintensity, FLAIR fluid-attenuated inversion recovery imaging.

All subjects underwent 3.0-Tesla brain MRI (Intera; Philips Medical Systems, Best, Netherlands), including susceptibility-weighted imaging, fluid-attenuated inversion recovery imaging (FLAIR), and T1-/T2-weighted imaging, obtained with the spin-echo technique. The repetition time for the T2-images was 3000 ms and the echo time was 15–90 ms. The corresponding parameters for the T1-weighted images were 300 and 15 ms, respectively. The slice thickness was 5 mm without an interslice gap. The imaging protocol for MRA was the three-dimensional time-of-flight method. All the MRI scans were reviewed by the same neurologist who was kept “blind” to the clinical data (Fig. 1).

On the T1-weighted axial, T2-weighted axial, and FLAIR images, periventricular WMHs (PVHs) and deep WMHs (DWHs) were separately evaluated as proposed by the Clinical Research Center for Dementia of South Korea [26]. The severity of DWHs was rated according to largest diameter, with the categories D1 (<10 mm), D2 (≥10 and <25 mm), and D3 (≥25 mm). The PVHs were rated as P1 if the cap or band was less than 5 mm, P2 if the cap or band was between 5 and 10 mm, and P3 if the cap or band was 10 mm or greater. By modifying prior criteria [27], we added grade 0 (absence; D0 or P0) to the individual ratings of PVHs and DWHs, and severity of total WMHs was reclassified as none (grade 0), minimal (grade 1), moderate (grade 2), or severe (grade 3) [26]. CMBs were defined as focal areas with very low signal intensities that measured less than 10 mm on susceptibility-weighted imaging. Cerebral microbleed mimics such as calcification, cerebral venules, and blood vessels, and cavernous malformations were not counted as instances of cerebral microbleed. Signal voids caused by sulcal vessels or symmetrical calcification in the basal ganglia, the choroid plexus, or pineal calcification were excluded [28, 29]. Lacunes were defined as small lesions (3–15 mm in diameter) with high signal intensities on T2- and low signal intensities on T1-weighted images or the perilesional halo on fluid-attenuated inversion recovery images [30]. The numbers of lacunar infarcts and cerebral microbleeds were also counted. In the present study, the MRI criteria of subcortical vascular dementia (SVaD), which included counts of microbleeds added from lacunes, were used as nominal dependent variables in logistic regression analyses, incorporating modification of the brain imaging criteria of subcortical ischemic vascular dementia proposed by Erkinjuntti et al. [31]. Medial temporal atrophy was rated on a five-point scale (0–4 points) on T1-weighted coronal images based on the width of the coronal fissure and the temporal horn and height of the hippocampal formation [32].

### Ambulatory BP monitoring

Automated 24-hour BP recording instruments (TM-2430; A&D, Tokyo, Japan) were used to measure BP every 30 min during the daytime (8 am to 11 pm) and every 60 min at night (11 pm to 8 am). The following parameters were evaluated: average sBP and diastolic BP (dBP) for the daytime, nighttime, and 24-hour periods. Mean sBP, mean dBP, and SDs of both were collected over 24 hours. According to recently issued guidelines, subjects could be classified according to BP characteristics and dipper status [33]. The ABP values were dichotomized into low and high groups according to the following respective conditions daytime 24-hour sBP (≤130 and >130 mmHg), 24-hour dBP (≤80 and >80 mmHg), daytime sBP (≤135 and >135 mmHg), daytime dBP (≤85 and >85 mmHg), nighttime sBP (≤120 and >120 mmHg), and nighttime dBP (≤70 and >70 mmHg) [34, 35]. Subjects were also divided into three dipper status categories for nocturnal BP: dippers, nondippers, and reverse dippers, defined as a difference in mean BP between daytime and nighttime hours greater than 10%, 0%–10%, and less than 0%, respectively [36, 37]. Separately, nighttime sBP variability and 24-hour pulse pressure could be divided into two categories as follows: low (≤10.8 mmHg) and high (>10.8 mmHg) sBP variability and low (≤53 mmHg) and high (>53 mmHg) pulse pressure, respectively [9, 38]. Among ABPM profiles, use of “Ambulatory Does Prediction Valid” parameters has been suggested from a practical standpoint and include average ambulatory BP, nocturnal dipping pattern, 24-hour pulse pressure, and variability of nighttime sBP [9].

### Statistical analysis

Group differences in demographic characteristics, cognitive and MRI findings, and ABPM profiles were compared by analysis of covariance and the χ^2^ test, as appropriate, after adjusting for age, sex, years of education, and body mass index (BMI). Moreover, multiple comparisons were performed by using the Benjamini–Hochberg correction. Partial correlation analyses were performed to examine the association between cognitive and MRI findings of cSVD, such as severity of WMH, numbers of lacune and cerebral microbleeds, and ABPM profiles, after controlling for the factors. Finally, multivariate logistic regression analyses for dependent variables of (1) dementia or not and (2) MRI criteria of SVaD or not were performed with independent variables of dichotomized ABPM profiles of high or low BP, nocturnal dipping status, high or low nighttime sBP variability, and high or low 24-hour pulse pressure after adjusting for age, sex, education, BMI. A *p* value less than 0.05 was considered statistically significant, and all tests were two-tailed. The data were analyzed using the Statistical Package for the Social Sciences version 15.0 software program (IBM Corp., Armonk, NY, USA).

## Results

### Characteristics of the subjects

A total of 174 subjects were included in this study. The mean age of the total study population was 75.36 ± 7.13 years, with significant differences between the three study groups (70.88 ± 6.51 years in the SCD group, 73.95 ± 0.85 years in the MCI group, and 76.99 ± 7.13 in the DAT group; *p* = 0.001). Subjects with DAT had 5.35 ± 4.72 years of education, while those with MCI had 6.78 ± 4.87 years and those with SCD had 9.53 ± 4.81 years. Duration of education was significantly different between the groups, adjusted for age (*p* = 0.034). Even after adjusting for age and years of education, MMSE score, clinical dementia rating (CDR), and CDR-sum of boxes (CDR-SB) significantly differed across the three subject groups (all, *p* < 0.001). After adjusting for age, no MRI and 24-hour BP variables except medial temporal atrophy varied between the three study groups. Table 1 lists the demographic data, while Table 2 is MRI findings for each type of subcortical ischemic lesion and 24-hour BPs for the three study groups.

**Table 1:** Demographic characteristics of the three groups.

	SCD	MCI	DAT	Total	*p* value	Post hoc
	(*n* = 17)	(*n* = 59)	(*n* = 98)	(*n* = 174)		
Age (years)	70.88 ± 6.51	73.95 ± 0.85	76.99 ± 7.13	75.36 ± 7.13	0.001	SCD vs. DAT, MCI vs. DAT
Sex (M:F)	8:9	17:42	30:68	55:119	0.344	
Education (years)	9.53 ± 4.81	6.78 ± 4.87	5.35 ± 4.72	6.25 ± 4.92	0.034	SCD vs. DAT
BMI	23.80 ± 3.47	24.29 ± 2.81	23.05 ± 2.96	23.54 ± 3.00	0.042	MCI vs. DAT
MMSE	27.29 ± 2.23	24.27 ± 3.22	17.03 ± 5.77	20.51 ± 6.23	<0.001^a^	SCD vs. DAT, MCI vs. DAT
CDR	0.47 ± 0.12	0.49 ± 0.07	1.13 ± 0.63	0.85 ± 0.57	<0.001^a^	SCD vs. DAT, MCI vs. DAT
CDR-SB	1.29 ± 0.77	1.68 ± 0.85	6.10 ± 3.66	4.13 ± 3.59	<0.001^a^	SCD vs. DAT, MCI vs. DAT
Medical conditions						
Diabetes	3	17	30	50	0.547	
Dyslipidemia	10	27	56	93	0.335	
Smoking	7	9	26	42	0.060	

Values are presented as means (and standard deviations) or raw numbers of patients.

*SCD* subjective cognitive decline, *MCI* mild cognitive impairment, *DAT* dementia of Alzheimer’s type, *BMI* body mass index, *MMSE* mini-mental state examination, *CDR* clinical dementia rating, *CDR-SB* clinical dementia rating—sum of boxes.

^a^Analyses were performed using analysis of covariance, adjusted for age, sex, years of education, and body mass index. Multiple comparisons were performed by using the Benjamini–Hochberg correction.

**Table 2:** Comparisons of MRI findings and ABPM profiles between the three groups.

	SCD	MCI	DAT	Total	*p* value	Post hoc
	(*n* = 17)	(*n* = 59)	(*n* = 98)	(*n* = 174)		
MRI						
PVH	1.47 ± 1.12 (0–3)	1.88 ± 1.04 (0–3)	2.15 ± 0.96 (0–3)	1.99 ± 1.02 (0–3)	<0.001^a^	SCD vs. DAT
DWH	0.76 ± 0.56 (0–3)	1.23 ± 0.91 (0–3)	1.26 ± 0.81 (0–3)	1.20 ± 0.83 (0–3)	0.025^a^	
WMH	1.06 ± 0.66 (0–2)	1.39 ± 0.74 (0–3)	1.51 ± 0.75 (0–3)	1.43 ± 0.75 (0–3)	0.002^a^	
Lacune	0.12 ± 0.33	0.65 ± 1.20	0.68 ± 1.51	0.61 ± 1.34	0.549^a^	
Cerebral microbleeds	0.06 ± 0.24	0.34 ± 1.16	1.11 ± 3.99	0.74 ± 3.09	0.378^a^	
Medial temporal atrophy	0.24 ± 0.44 (0–1.5)	0.74 ± 0.95 (0–3.5)	1.33 ± 1.13 (0–4)	1.02 ± 1.08 (0–4)	<0.001^a^	SCD vs. DAT, MCI vs. DAT
ABPM profile						
sBP (24-hour)	124.85 ± 10.60	127.93 ± 12.85	128.61 ± 14.86	128.01 ± 13.81	0.812	
Low:High	13:4	34:25	55:43	102:72	0.491	
dBP (24-hour)	74.31 ± 5.49	76.53 ± 9.01	75.61 ± 8.49	75.70 ± 8.44	0.223	
Low:High	16:1	41:18	71:27	128:46	0.07	
sBP (daytime)	125.67 ± 10.49	129.36 ± 12.81	129.97 ± 14.97	129.34 ± 13.87	0.732	
Low:High	16:1	41:18	62:36	119:55	0.089	
dBP (daytime)	74.37 ± 5.60	77.73 ± 9.28	76.88 ± 8.91	76.92 ± 8.78	0.246	
Low:High	17:0	48:11	80:18	145:29	0.081	
sBP (nighttime)	122.51 ± 12.62	124.12 ± 15.15	124.67 ± 16.56	124.27 ± 15.68	0.971	
Low:High	8:9	25:34	40:58	73:101	0.976	
dBP (nighttime)	70.33 ± 6.57	73.24 ± 10.19	71.73 ± 8.98	72.10 ± 9.21	0.296	
Low:High	7:10	22:37	40:58	69:105	0.791	
Dipping	3.95 ± 5.72	4.85 ± 7.39	5.23 ± 7.37	4.98 ± 7.21	0.947	
Low:High (N:R)	2:15 (11:4)	17:42 (29:13)	24:74 (54:20)	43:131 (94:37)	0.322	
Nighttime sBP variability	11.13 ± 4.24	12.57 ± 4.76	12.35 ± 5.58	12.31 ± 5.19	0.675	
Low:High	9:8	25:34	49:49	83:91	0.414	
24-hour pulse pressure	51.55 ± 9.19	51.39 ± 7.94	53.00 ± 11.05	52.31 ± 9.90	0.836	
Low:High	11:6	35:24	54:44	100:74	0.986	

Values are presented as means (and standard deviations) or raw numbers of patients (in low and high categories). In addition, MRI visual rating scales such as PVH, DWH, and WMH are presented as ranges in parentheses, as categorial variables.

*MRI* magnetic resonance imaging, *ABPM* ambulatory blood pressure monitoring, *SCD* subjective cognitive decline, *MCI* mild cognitive impairment, *DAT* dementia of Alzheimer’s type, *PVH* periventricular white matter hyperintensity, *DWH* deep white matter hyperintensity, *WMH* white matter hyperintensity, *sBP* systolic blood pressure, *dBP* diastolic blood pressure, *high (N:R)* high (nondipper: reverse dipper).

^a^Analyses were performed using analysis of covariance, adjusted for age, sex, education, and body mass index. Multiple comparisons were performed by using the Benjamini–Hochberg correction.

### Correlation among clinical, imaging, and ABPM findings

**Fig. 2:**
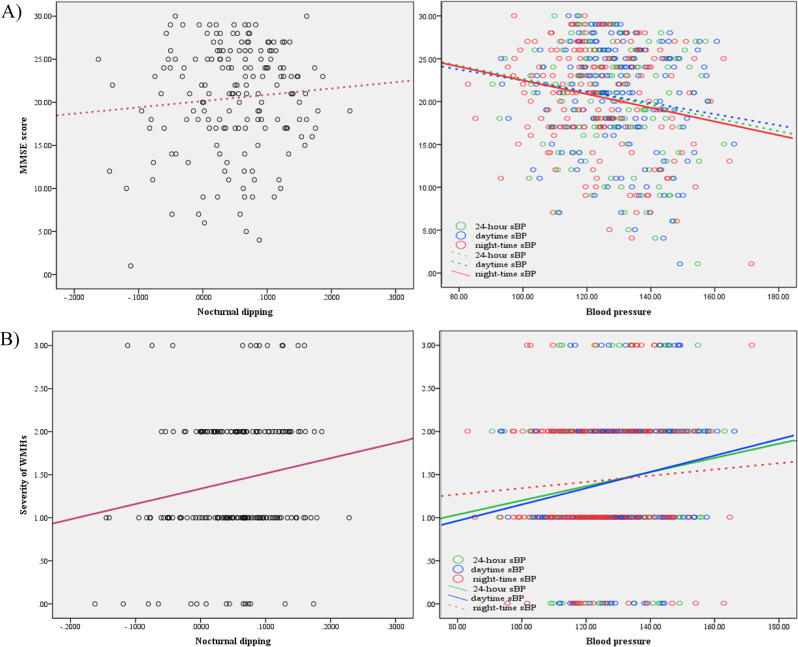
Correlation of MMSE score (A) and WMH severity (B) with ABPM profile. Partial correlation analyses, adjusted for age, sex, education, and body mass index, show the associations of MMSE score (**A**) with nocturnal dipping (*r* = 0.092; *p* = 0.243), 24-hour sBP (*r* = −0.151; *p* = 0.055), daytime sBP (*r* = −0.125; *p* = 0.112), and nighttime sBP (*r* = −0.187; *p* = 0.017) and the associations of WMHs (**B**) with nocturnal dipping (*r* = 0.106; *p* = 0.039), daytime sBP (*r* = 0.171; *p* = 0.030), nighttime sBP (*r* = 0.109; *p* = 0.166), and 24-hour sBP (*r* = 0.158; *p* = 0.044). MMSE mini-mental state examination, WMH white matter hyperintensity, ABPM ambulatory blood pressure monitoring, sBP systolic blood pressure.

Figure 2 presents partial correlations between the study’s key variables. The 24-hour sBP was negatively correlated with MMSE score, with a borderline significance (*r* = −0.151; *p* = 0.055), and positively correlated with the severity levels of DWH (*r* = 0.177; *p* = 0.024) and WMH (*r* = 0.158; *p* = 0.044). Meanwhile, daytime sBP was positively correlated with the severity levels of DWH (*r* = 0.184; *p* = 0.019) and WMH (*r* = 0.171; *p* = 0.030), but not with MMSE score (*r* = −0.125; *p* = 0.112). Nighttime sBP was correlated negatively with MMSE score (*r* = −0.187; *p* = 0.017) and positively with severity of DWH (*r* = 0.145; *p* = 0.065), with a borderline level of significance. Finally, the value of nocturnal dipping was correlated with CDR (*r* = −0.162; *p* = 0.039) and CDR-SB (*r* = −0.166; *p* = 0.035), and WMH severity (*r* = 0.106; *p* = 0.039), but not with MMSE score (*r* = 0.092; *p* = 0.243). Nighttime sBP variability and 24-hour pulse pressure were not correlated with any MRI findings.

### Associations of 24-hour blood pressure values with dementia and MRI criteria of SVaD

The odds ratios (ORs) for dichotomized ABPM profiles for dementia and the MRI criteria of SVaD were evaluated in older subjects with cognitive complaints. Only higher daytime sBP independently correlated with dementia v[OR = 3.408; 95% confidence interval (CI): 1.004–11.571; *p* = 0.049]. Also, higher daytime sBP showed a correlation with the MRI findings of SVaD (OR = 8.937; 95% CI: 1.034–77.261; *p* = 0.047). Other ABPM profiles, when dichotomized, did not correlate with dementia or not or with MRI criteria of SVaD or not, as suggested by multivariate logistic regression analysis.

## Discussion

This study demonstrated that, although there were no differences in ABPM profiles among the three groups (SCD, MCI, and DAT), a higher BP, especially a higher sBP, correlated positively with cognitive dysfunction and severity of WMH in older adults. Also, decreases in nocturnal dipping correlated with functional disability as measured by CDR score and had borderline significances with both MMSE score and WMH severity. Multivariate analysis using a logistic regression model showed that only higher daytime sBP was an independent predictor of dementia and MRI findings of SVaD. Among the various ABPM profiles, a higher BP, especially a higher sBP, may be considered more important for predicting clinical and MRI findings.

Previous studies found that hypertension, especially sBP, was closely related to WMH and cognition [39–44], and the mean sBP was higher in patients with cSVD than in controls [45, 46] in prior ABPM research. Studies also showed that sBP but not dBP was associated with cSVD burden [47], which might be the result of the greater impact of sBP than dBP on vascular diseases [48]. The present study also reported that sBP, rather than dBP, was correlated with cognitive and MRI findings of cSVD.

The potential pathophysiological mechanisms underlying the association between BP level and cSVD burden are complex and not completely understood. Increased permeability of small-vessel walls and blood–brain barrier has been suggested to contribute to development of cSVD and has been reported to be associated with microvascular endothelial-cell and tight-junction damage [1, 49]. A higher BP level would lead to greater mechanical stress on the vessel wall, which progresses to endothelial injury and arterial stiffness [50, 51]. Therefore, it is reasonable to assume that a higher BP would contribute to development of cSVD via endothelial-cell damage. In addition, ischemic hypoperfusion is thought to be involved in the pathogenesis of cSVD [1].

Measurement of nighttime BP yielded additional prognostic data in terms of all-cause mortality and cardiovascular events. Nighttime BP, adjusted for daytime BP, independently predicted total, cardiovascular, and noncardiovascular mortality rates [52]. In particular, available data suggest that nighttime BP is more important than daytime BP in predicting subclinical organ damage and cardiovascular outcome, particularly in individuals whose nocturnal (sleep) BP remains high [52], but the reason for these differences is unclear. Our results also showed that nighttime sBP and 24-hour sBP were associated with MMSE score and DWH severity, although we did not study whether daytime or nighttime BP is more important.

AD patients also exhibit disturbed day–night BP dipping [53]. Contrary to previous research, this study did not show group differences in dipping status; however, levels of nocturnal dipping significantly correlated with CDR and had borderline significance with WMHs and MMSE. The more blunted or abolished nocturnal fall in BP subjects had, the more severe cognitive impairment and white matter changes they showed. One possible explanation is lack of involvement of controls in this study. Moreover, like all categorizations of continuous variables, the dipper–nondipper classification has been criticized because it implies arbitrary dichotomization of a continuous variable. However, such a classification approach appears useful from a clinical standpoint since several reports from independent centers have shown that not only left ventricular hypertrophy [54], but also silent cerebrovascular disease [55] and microalbuminuria [56] were more frequently observed in subjects with a blunted or abolished decrease in BP from day to night than in those with normal day–night BP differences. In patients with Binswanger’s disease, a prominent loss of diurnal BP rhythm was recently observed [45]. The same researchers also found that nondippers were more prevalent in the cSVD group than in the control group. The precise relationship between subcortical small-vessel lesions and loss of dipping is controversial, with some researchers [20] suggesting that dipping status is unrelated to cerebral pathology. However, many other studies have found that nondipping is closely correlated with possible hypertensive target-organ damage, and that nondipping and reverse-dipping statuses are more common among patients with subcortical small-vessel ischemia [12, 19, 44, 46]. O’Brien et al. [13] reported a more frequent history of stroke in nondippers than in dippers.

The present study did not show the associations of BP variability and pulse pressure with cSVD. Although research has suggested that sBP variability and pulse pressure are significantly higher in SVaD patients [57], indicating a possible association with loss of vasomotor reactivity in advanced small-vessel lesions, there remains considerable controversy regarding the relationships between BP variability or pulse pressure and ischemic white matter lesions and cognition. It is noteworthy that van Boxtel et al. [20] found no conclusive evidence of a connection between diurnal BP variation and early target-organ damage in the brain. Moreover, when 24-hour sBP and 24-hour pulse pressure were forced into the same model, only 24-hour sBP achieved significance, although 24-hour pulse pressure bordered on statistical significance [58]. While pulse pressure was the dominant predictor of cardiac events, for cerebrovascular events, mean BP, not pulse pressure, was the major independent predictor [59].

This study had several limitations. First, the classification and relationships among SCD, MCI, and DAT were not approved or confirmed by biomarkers such as those on amyloid positron emission tomography. However, we tried to reduce selection bias by only including patients who had fulfilled the clinical diagnostic criteria for each disease [22–25]. In addition, we did not include controls; meanwhile, our enrollment covered patients with a comprehensive spectrum of memory disorders, including SCD and MCI. Second, the number of participants is relatively small. Especially, there are only 17 people with SCD, which might influence the study results. Women were only 52.9% (9/17) in SCD, contrary to MCI (71.2%) and DAT (69.4%). Moreover, small effect size and power also could be overcome with larger sample size. Effect size presented as partial eta squared was 0.116 in comparison of medial temporal atrophy (observed power 0.982). Other MRI and ABPM findings were below 0.06. Also in partial correlation analysis, all of the correlation coefficient was within 0.30. Third, most hypertensive patients were taking antihypertensive medications. Therefore, these data must be carefully interpreted as antihypertensive medications can contribute to BP and BP variability. In addition, we did not assess any other mechanisms that might have played a pathophysiological role in BP and BP variability. For example, cholinesterase inhibitors can reduce heart rate variability and affect sleep and arousal [60]. Especially, women included in this study might even be at the different stages of the menopause transition and, if any, this directly effects estrogen levels that has an influence on vascular function and subsequently BP [61]. Estrogen replacement therapy is also associated with a decreased risk for dementia, but not in women already diagnosed with AD [62, 63]. In addition, although major psychiatric diseases were excluded, we did not investigate whether participants took benzodiazepine, anticholinergics, antidepressants, etc., which can also have effects on cognition. Next, our findings can also be explained by reverse causality. People with greater cognitive impairment such as MCI and AD are also more prone to other cerebrovascular risk factors that would influence the associations between ABPM profiles and cognitive and MRI findings. That is, higher BP could be just a risk marker of the influence of an overall worsening of health and quality of life, which in turn affects cognition. Further studies with larger sample size and measurements of various vascular risk factors including obesity, diet, and physical activity and a prospective follow-up investigation are needed to clarify the difference between mild AD patients and advanced AD patients and to assess longitudinal changes during disease progress.

Based on the results of this study, it is suggested that the ABPM is useful for measurements of cognitive and imaging findings related to cSVD and that ABPM profile might be predictive of progression of cSVD. Modulating the loss of nocturnal dipping could also help prevent dementia related to advanced cSVD, while simultaneously lowering the BP.

### Summary

#### What is known about this topic

- Cerebral small-vessel disease (cSVD) is a common vascular risk factor of dementia and has an additive effect on cognitive decline in patients with Alzheimer’s disease (AD).
- Although the pathogenesis of cSVD is not completely understood, hypertension is a well-known major risk factor.
- Ambulatory blood pressure (BP) monitoring (ABPM) is used to study BP under normal living conditions as it provides a reliable estimate of the habitual diurnal BP rhythm, which may be used to independently predict hypertension-related complications.

#### What this study adds

- ABPM is useful for measurements of cognitive and imaging findings related to cSVD.
- Among ABPM profiles, a higher BP, especially a higher systolic BP, may be the most important for clinical and magnetic resonance imaging findings of cSVD. A higher systolic BP correlated with Mini-Mental State Examination (MMSE) scores and severity of white matter hyperintensities (WMHs).
- Decreases in nocturnal dipping correlated with clinical dementia rating (CDR) scores and had borderline significances with both MMSE score and WMH severity.
